# “You would not be in a hurry to go back home”: patients’ willingness to participate in HIV/AIDS clinical trials at a clinical and research facility in Kampala, Uganda

**DOI:** 10.1186/s12910-020-00516-z

**Published:** 2020-08-24

**Authors:** Deborah Ekusai Sebatta, Godfrey Siu, Henry W. Nabeta, Godwin Anguzu, Stephen Walimbwa, Mohammed Lamorde, Badru Bukenya, Andrew Kambugu

**Affiliations:** 1 Infectious Diseases Institute, Makerere University Kampala, Kampala, Uganda; 2 Department of Child Health and Development Centre, Makerere University Kampala, Kampala, Uganda; 3 University of Louisville, School of Medicine, Louisville, KY USA; 4 Department of Social work and Social Administration, Makerere University Kampala, Kampala, Uganda

**Keywords:** Clinical trials, Willingness to participate, Perceived benefit, Compensation, Barriers

## Abstract

**Background:**

Few studies have examined factors associated with willingness of people living with HIV (PLHIV) to participate in HIV treatment clinical trials in Sub-Saharan Africa. We assessed the factors associated with participation of PLHIV in HIV treatment clinical trials research at a large urban clinical and research facility in Uganda.

**Methods:**

A mixed methods study was conducted at the Infectious Diseases Institute (IDI), adult HIV clinic between July 2016 and January 2017. Data were collected using structured questionnaires, focused group discussions with respondents categorised as either participated or never participated in clinical trials and key informant interviews with IDI staff. A generalized linear model with a logit link function was used for multivariate analyses while the qualitative data were summarized using a thematic approach.

**Results:**

We enrolled a total of 202 and analysed 151 participants, 77 (51%) of whom were male with mean age of 41 years. The majority 127 (84%) expressed willingness to participate in treatment clinical trials if given an opportunity. At bivariate analysis, willingness to participate was significantly associated with respondents’ perception of a satisfactory compensation package (*P*-value < 0.002, 0.08–0.56), special status accorded (P-value < 0.001, 0.05–0.39) and belief that their health status would improve (*P*-value< 0.08, 0.03–0.58) while on the clinical trial. At multivariate analysis, a satisfactory compensation package (*P*-value< 0.030, 0.08–0.88) and special status accorded in clinical trials (*P*-value< 0.041, 0.01–0.91) remained significant. The qualitative data analysis confirmed these findings as participants valued the privilege of jumping the clinic waiting queues and spending less time in clinic, the wide range of free tests offered to trial participants, unrestricted access to senior physicians and regular communication from study team. Additionally, free meals offered during clinic visits meant that participants were *not in a hurry to go back home*. Barriers to participation included the perception that new drugs were being tested on them, fear of side effects like treatment failure and the uncertainty about privacy of their data.

**Conclusion:**

We found overwhelming willingness to participate in HIV treatment clinical trials. This was largely extrinsically influenced by the perceived material and health-related benefits. Investigators should pay attention to participants’ concerns for benefits which may override the need to understand study procedures and risks.

## Background

The burden of HIV in Uganda remains high with an estimated prevalence of 6.2%. This corresponds to approximately 1.2 million people living with HIV in Uganda [1]. In the general population, new annual HIV infections declined from 160,000 to 95,000 between 2010 and 2014. However, new infections remain unacceptably high ([2], p. 1) with significant variability especially among key populations [3]. While antiretroviral therapy is effective for long term suppression, research questions remain with respect to strategies for prevention of HIV transmission, strategies to retain patients in care, and optimal treatment regimens that are effective [4]. Addressing these questions will require additional studies including well-designed clinical trials.

Over the past decade, the number of HIV treatment clinical trials (CT) in Africa has increased [5]. This increase has led to emergence of ethical concerns about people living with HIV participating in drug treatment clinical trials in Africa.

Studies conducted in high-income countries and in low-income countries on the willingness to participate in clinical trials have reported varied results. In the United Kingdom and United States, poor recruitment rates have been reported and attributed to non-approachable clinicians, fear and mistrust of researchers, patient attitudes, stringent eligibility criteria and lack of knowledge about clinical research [6–8]. Studies in sub-Saharan Africa showed that participants tend to exhibit a moderate willingness to participate in HIV vaccine trials. This is largely associated with perceived benefits, necessity, safety and understanding of clinical trials [5, 9]. The impression that there is a better understanding of clinical trials in Africa and a lack of knowledge about clinical research in high-income countries is rather contentious. It is possible that individuals in sub-Saharan Africa are more willing to participate in clinical trials because of compensation packages and research benefits and not because they understand clinical trials better than individuals in the high-income countries do. The barriers to participation in clinical trials include low literacy levels, stigma, discrimination and fears, convenience, and compensation and receipt of medical care [10–12].

Understanding why people volunteer to participate in treatment clinical trials will provide important information that can improve the recruitment process, inform public health policy and practice by aiming at improving the quality of clinical trials. A qualitative study that investigated how volunteers taking part in HIV clinical trials in central Uganda perceived informed consent, trial procedures, study information and their interaction with study staff, found that during the consent process, volunteers paid more attention to procedures requiring biological tests than to study design issues [12]. The sustained participation and cooperation of patients is essential for the success of a clinical trial (Agnes [13]).

This study aimed to understand whether people are intrinsically or extrinsically motivated to participate in HIV treatment clinical trials. We hypothesised that there is a relationship between perceived benefit/ compensation and willingness to participate in clinical trials.

We examined factors, perceptions and motivations associated with participation in drug treatment clinical trial research. We sought to determine the relationship between perceived benefit/ compensation and participants’ willingness to participate in HIV/ AIDS treatment clinical trials at IDI and to establish other factors that could affect their willingness to participate in treatment trials.

## Methods

**Fig. 1:**
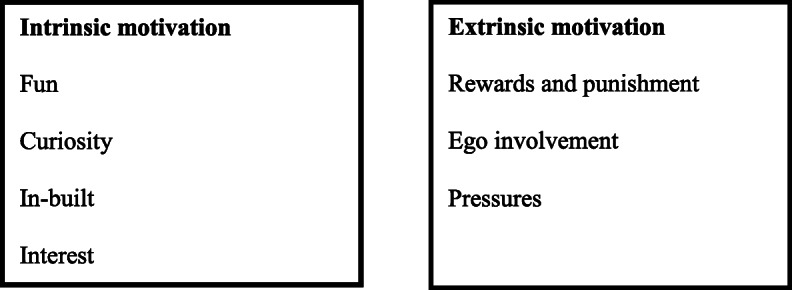
Intrinsic and Extrinsic theory of Motivation

An intrinsic and extrinsic theory of motivation was utilised in this study [14].

(Fig. 1). Intrinsic motivation refers to doing something for its own sake, interest or for the pure enjoyment of a task. When clinical trial participants are active, curious, and eager to learn and engage in the studies, they display intrinsic motivation [14]. Extrinsic motivation refers to doing something in order to attain some external goal or meet some externally imposed constraint [15].

### Design and sample

#### Quantitative sample

We conducted a cross-sectional survey using a mixed methods approach among patients receiving care at IDI. Two categories of respondents were identified, category one: those who were either enrolled in an on-going treatment clinical trial or had concluded participation within the last 3 years because of their recent experience; category two: those who had never participated in any treatment clinical trial.

Our operational definition of willingness to participate was “the expressed desire by a respondent to participate in a clinical trial, if given an opportunity”. Respondents who had participated in clinical trials before the start of our study were classified as having expressed willingness to participate in clinical trials. We also assumed that they would still be interested in participating in CTs after 3 years. Respondents with no prior experience with clinical trials were asked if they were interested in participating in future CTs and those with a positive response were categorised as having expressed willingness to participate. However, only respondents with knowledge about clinical trials were assessed for willingness to participate which was calculated using the following formula [16]:

$$ {\displaystyle \begin{array}{l}\mathrm{Willingness}\ \mathrm{to}\ \mathrm{participate}=\\ {}\left(\begin{array}{l}\mathrm{Number}\ \mathrm{of}\ \mathrm{people}\ \mathrm{that}\ \mathrm{had}\ \mathrm{ever}\ \mathrm{participate}\mathrm{d}\ \mathrm{in}\ \mathrm{clinical}\ \mathrm{trials}+\\ {}\mathrm{Never}\ \mathrm{participate}\mathrm{d}\ \mathrm{but}\ \mathrm{willing}\ \mathrm{if}\ \mathrm{approached}\end{array}\right)\\ {}\div \mathrm{Total}\ \mathrm{population}\end{array}} $$

The operational definition of a satisfactory compensation package included incentives like compensation for time and transport reimbursement that were reasonable and acceptable to the study participants.

A sample size of 384 participants was estimated using Kish and Leslie formula. Quantitative research participants were selected from the IDI adult clinic. Systematic sampling was performed to obtain a true representative sample of the HIV population attending this clinic and every third person was approached using a detailed information sheet.

#### Qualitative sample

A sample of 45 participants were selected for the qualitative arm of the study and they participated as either key informants or focus group discussants. The participants details are described in the results section. A total of 5 key informants were purposively selected to include health providers and a peer educator for their knowledge and involvement in HIV treatment clinical trials. Focus group participants were systematically selected from an electronic data base, and every third participant on the list was contacted until a total of 40 was realized.

### Setting

The study was conducted at the Infectious Diseases Institute (IDI) adult HIV clinic, Kampala, Uganda between July 2016 and January 2017. Established within Makerere University College of Health Sciences, the Institute was opened in 2002. IDIs adult HIV clinic currently provides care and treatment services to over 80,000 people living with HIV in urban and rural settings in Uganda and has completed 33 clinical trials. Study procedures, compensation and benefits vary across studies. In pharmacokinetic studies within the institute, every clinical trial participant is provided compensation in monetary terms equivalent to UGX 30,000–50,000 shillings (8-13USD) for time spent during a clinic visit and an additional 10,000–30,000 shillings (2–8 USD) as transport reimbursement. Additional non-monetary benefits include special status accorded like enhanced follow-ups by research teams, jumping patient queues, access to medical specialists, vital tests and free meals during clinic visits.

#### Inclusion and exclusion criteria

Participants were eligible if they provided informed consent, were at least 18 years of age, had a confirmed HIV positive test and were attending IDI clinic for HIV care.

Participants were excluded if they were very sick, unable to provide informed consent and if they had participated in a clinical trial that ended more than 3 years prior to consenting to this study.

### Data collection

#### Qualitative data

The qualitative data were collected using focus group discussions (FGDs) and key informant interviews (KII). An FGD guide was used during the discussions to explore participants motivations and barriers to participating in HIV treatment clinical trial research. Four FGDs were held each comprising of 7–12 participants with either respondents who had participated in drug treatment clinical trials or those who had never participated in any clinical trials. Luganda, the most widely spoken language in Kampala was used to conduct the focus group discussions and English was used to conduct the key informant interviews in a private room on the IDI premises.

Five KIIs were conducted using an interview guide to obtain information from experienced IDI staff members to capture their views on the willingness of people living with HIV (PLHIV) to participate in HIV treatment clinical trials at the IDI. Additionally, the KIIs sought to understand how institutional factors influence PLHIV to participate in HIV treatment CTs. The first author and two research assistants conducted the interviews. The research assistants were trained in good clinical practice and on how to conduct FGDs and KIIs. The interview guides were piloted and revised prior to the full data collection process. Audio recorders were used to capture the FGDs and KII interviews to ensure all information was collected. The recorded interviews were transcribed in the language they were conducted and where necessary translated into English.

#### Quantitative data

The Interviewer administered structured questionnaires which lasted approximately 30 min. The questionnaires were pre-tested to ensure question clarity and length. Four research assistants were trained on the research protocol, data collection tools and interviewing techniques before conducting any interviews. The interviews were conducted in English, and Luganda which is the most widely spoken language in the study area. The questionnaire included basic demographic information and experience with HIV treatment clinical trials. Awareness of clinical trials was assessed by asking, “Have you ever heard of clinical trials?” The participants who had never heard of clinical trials were excluded from the interview. Participants were then asked; From where did you hear about clinical trials? What is your understanding of clinical trials? What is your attitude to participating in clinical trials? How do you perceive participation in clinical trials?

### Ethical statement

Ethics approval was obtained from the Mulago Hospital Research Ethics Committee (MREC 991) in June 2016. Additional approvals were obtained from the Uganda National Council for Science and Technology (HS SS 5013) in July 2016. All participants signed an informed consent sheet and confidentiality was maintained by anonymizing participants’ identifiers at all stages of data collection and reporting.

### Data manage2ment and statistical analyses

The data were entered into a computer using EPIDATA Version 3.02 and later exported to STATA for analysis. Descriptive statistics for background, demographics were summarized using frequencies, median, means and proportions. Univariate and bivariate analyses were conducted to investigate relationships between these data and awareness of clinical trials, perceptions of clinical trials, and willingness to participate in clinical trials. Associations between the participants’ characteristics and willingness to participate in a clinical trial were tested using the chi-square test statistic (χ^2^ test) for categorical variables, independent sample t-test and ANOVA for continuous variables. Multivariate analyses were conducted using a generalized linear model (GLM) with a logit link function.

#### Qualitative data analysis

The FGDs and key informant interviews were transcribed by an experienced research assistant. Thematic analysis was performed manually [17]. This involved the first author carrying out multiple readings of the scripts to understand the data and subsequently coding the data using pre-defined areas of analysis based on the objectives of study. However, the process was open to identifying new themes from the data, which were integrated into the pre-existing themes.

## Results

**Fig. 2:**
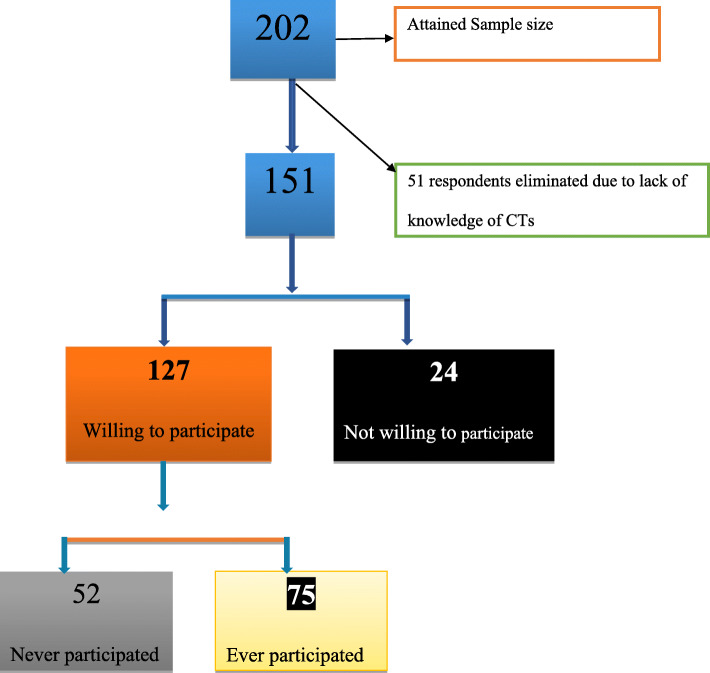
Distribution of sample

We consented 202 participants, excluded 51 who had no knowledge of clinical trials and analysed data of 151 participants. (Fig. 2).

### Characteristics of the participants

#### Quantitative

We analysed data for 151 adult participants (Table 1), of whom 77(51%) were male. Only 127 (84%) met the operational definition of willingness to participate in clinical trials and 75(59%) of whom had ever participated in clinical trials. Of the respondents enrolled, 75(50%) had participated in previous trials and 76(50%) had not been previously enrolled in any clinical trials but expressed willingness to participate in such studies if given an opportunity. The mean age was 41.6 (SD 11.9). The majority subscribed to the Roman Catholic faith 56 (37%), 51 (33%) were married, 67(44%) had attained secondary-level education and 104(69%) were employed. Most of the participants were self-employed 52(50%) and earning an average income of Uganda Shillings (120,000–300,000UGX) (approx. 50–100 USD) per month.

**Table 1:** Description of Study population

Variables	Overall [***N*** = 151 (%)]	Willing to participate in CTS Yes [***n*** = 127(%)] No [***n*** = 24(%)]		P-value
**DEMOGRAPHICS**				
**Age; mean (st.dev)**	41.6 (11.9)	41.2 (11.7)	43.8 (13.1)	**0.322**^**a**^
**Sex**				**0.319**
Male	77 (51.0)	67 (52.8)	10 (41.7)	
Female	74 (49.0)	60 (47.2)	14 (58.3)	
**Marital status**				**1.000**
Married	91 (60.3)	76 (59.8)	15 (62.5)	
Separated	33 (21.8)	28 (22.1)	5 (20.8)	
Never married	11 (7.3)	9 (7.1)	2 (8.3)	
Widow/widower	16 (10.6)	14 (11.0)	2 (8.3)	
**BENEFITS OF PARTICIPATING IN CTS**				
**Improved health status**				**0.013**^**1**^
Yes	144 (95.4)	124 (97.6)	20 (83.3)	
No	7 (4.6)	3(2.4)	4 (16.7)	
**Special status**				**< 0.001**
Yes	128 (87.7)	113 (92.6)	15 (62.5)	
No	18 (12.3)	9 (7.4)	9 (37.5)	
**Develop new treatment**				**0.053**
Yes	129 (86.6)	112 (88.9)	17 (73.9)	
No	20 (13.4)	14 (11.1)	6 (26.1)	
**Future benefit**				**0.228**
Yes	132 (90.4)	114 (91.9)	18 (81.8)	
No	14 (9.6)	10 (8.1)	4 (18.2)	
**Compensate participants**				**1.000**^**1**^
Yes	133 (88.7)	111 (88.1)	22 (91.7)	
No	14 (9.3)	12 (9.5)	2 (8.3)	
Don’t know	3 (2.0)	3 (2.4)	0	
**Fairly compensated**				**0.002**
Yes	94 (65.2)	86 (71.1)	8 (34.8)	
No	25 (17.4)	19 (15.7)	6 (26.1)	
Don’t know	25 (17.4)	16 (13.2)	9 (39.1)	
**ULTIMATE BENEFICIARIES**				
**Pharmaceutical companies**				**0.935**
Yes	43 (28.5)	36 (28.4)	7 (29.2)	
No	108 (71.5)	91 (71.6)	17 (70.8)	
**Hospitals**				**< 0.001**
Yes	48 (31.8)	33 (26.0)	15 (62.5)	
No	103 (68.2)	94 (74.0)	9 (37.5)	
**Future patients**				**0.081**
Yes	110 (72.9)	96 (75.6)	14 (58.3)	
No	41 (27.1)	31 (24.4)	10 (41.7)	
**Encourage CTs participation**				**< 0.001**^**1**^
Yes	136 (95.1)	120 (100.0)	16 (69.6)	
No	7 (4.9)	0(0.0)	7(30.4)	
**BARRIERS**				
**Fear of CT drugs**				**0.167**
Yes	34 (22.5)	26 (20.5)	8 (33.3)	
No	117 (77.5)	101 (79.5)	16 (66.7)	
**Blood loss**				**0.201**^**1**^
Yes	20 (13.2)	19 (15.0)	1 (4.2)	
No	131 (86.8)	108 (85.0)	23 (95.8)	
**Pain**				**0.127**^**1**^
Yes	24 (15.9)	23 (18.1)	1 (4.2)	
No	127 (84.1)	104 (81.9)	23 (95.8)	
**Treatment failure**				**0.008**
Yes	19 (12.6)	12 (9.5)	7 (29.2)	
No	132 (87.4)	115 (90.5)	17 (70.8)	
**Death**				**0.036**^**1**^
Yes	9 (6.0)	5 (3.9)	4 (16.7)	
No	142 (94.0)	122 (96.1)	20 (83.3)	
**INSTITUTIONAL FACTORS**				
**Fair selection**				**0.082**^**1**^
Yes	123 (86.0)	106 (88.3)	17 (73.9)	
No	15 (10.5)	11 (9.2)	4 (17.4)	
I don’t know	5 (3.5)	3 (2.5)	2 (8.7)	
**Year Joined IDI**				**0.387**^**1**^
2002–2005	61 (40.4)	48 (37.8)	13 (54.2)	
2006–2010	39 (25.8)	34 (26.8)	5 (20.8)	
2011–2016	51 (33.8)	45 (35.4)	6 (25.0)	

P^1^ = fisherman’s exact p-value, P = Chi square p-value, P^a^ = ttest p-value.

### Factors associated with willingness to participate in clinical trials

#### Quantitative results

For participants in pharmacokinetic trials, receiving all meals (breakfast, lunch, dinner) was perceived as being a satisfactory incentive. A transport refund of 10,000 UGX (2.7USD) was deemed as the threshold for de-satisfaction which was a hindrance to CT participation. At bivariate analysis, we assessed the association between participants’ age, sex, perception of benefits including if they viewed the compensation package as satisfactory and willingness to participate in drug treatment clinical trials (Table 1). Gender, age, future benefit of clinical trials were not statistically significant and results are summarised in Table 1. The statistically significant factors for willingness to participate in clinical trials were: participants’ perception of a satisfactory compensation package (P-value = 0.002, 0.08–0.56), belief that they would have special status accorded to them (*P*-value = 0.001, 0.05–0.39), and belief that they would have improved health status (*P*-value = 0.08,0.03–0.58) if they participated in treatment clinical trials.

At multivariate analysis, a threshold of 0.25 was set and variables with *P*-values < 0.25 from the Univariate analysis were considered. A generalised linear model (GLM) was then adjusted at multivariate analysis. Age and sex were considered fixed factors from literature. We established two significant variables: participant perception that the compensation package was satisfactory (*P*-value < 0.030, 0.08–0.88) and special status accorded in clinical trials (P-value < 0.041, 0.01–0.91). The odds of willingness to participate in clinical trials among participants who believed that the compensation package was not satisfactory was 0.27 times that of participants who felt they were satisfactorily compensated (Table 2). Similarly, the odds of willingness to participate in clinical trials among those who believed that they would not have special status accorded to them was 0.11 times that of participants who thought they would have a special status in clinical trials. This means that perception of a satisfactory compensation package and special status accorded in clinical trials influenced willingness to participate, thereby proving our hypothesis.

**Table 2:** Showing factors associated with willingness to participate in HIV Clinical Trials

Variable	Unadjusted OR (95% CI)	P-value	Adjusted OR (95% CI)	P-value
Age	0.98 (0.95–1.02)	0.320	0.98 (0.94–1.03)	0.531
Sex				
Female	1.00		1.00	
Male	1.56 (0.65–3.78)	0.321	1.74 (0.52–5.84)	0.372
Improved health status				
Yes	1.00		1.00	
No	0.12 (0.03–0.58)	0.008	0.66 (0.04–12.44)	0.784
CTs have a future benefit				
Yes	1.00		1.00	
No	0.39 (0.11–1.39)	0.149	0.80 (0.10–6.56)	0.833
Satisfactory compensation package				
Yes	1.00		1.00	
No	0.22 (0.08–0.56)	0.002	0.27 (0.08–0.88)	0.030
Patients are beneficiaries of CTs				
Yes	1.00		1.00	
No	0.45 (0.18–1.12)	0.086	0.73 (0.22–2.44)	0.606
CTs used in the development of new drugs				
Yes	1.00		1.00	
No	0.35 (0.12–1.05)	0.061	5.52 (0.43–71.28)	0.190
Special status accorded				
Yes	1.00		1.00	
No	0.13 (0.05–0.39)	< 0.001	0.11 (0.01–0.91)	0.041
Side effects				
Yes	1.00		1.00	
No	0.20 (0.03–1.53)	0.120	0.25 (0.03–2.23)	0.215

### Qualitative results

We explored experiences of 45 participants, balanced by gender. A total of 40 participants took part in the FGDs and while 5 took part in key informant interviews; these included four health providers and a peer educator. Participation in the FGDs is presented in Table 3.

**Table 3:** Focus group discussions overview

FGD	Age range	No. of participants	Male	Female	CT participation
**1**	20–32	7	3	4	Never participated
**2**	35–50	12	5	6	Participated
**3**	27–33	11	3	9	Never participated
**4**	36–50	10	8	2	Participated
**Total**		40	19	21	

### Main themes identified

Content analysis identified four main themes from both the FGDs and KIIs. These are summarised in the table below (Table 4):

**Table 4:** Main themes identified

Topic	Main theme 1: Special Status	Main theme 2: Improved health status	Main theme 3: Meals	Main theme 4: Ultimate beneficiaries of CTs
**Perception of non-monetary benefits**	Sub-themes jumping the queue Seen by senior physicians Spending less time at the clinic Frequent Communication by study team Value of tests	Subthemes - condition of living Symptoms reduced Reduced number of visits to the clinic	Sub themes Breakfast Lunch supper	Sub themes -pharmaceutical companies Patients hospitals
**Perception of monitory benefits**	Main theme Compensation for time	Main theme Financial reimbursement		
	Sub theme Satisfactory compensation Un satisfactory compensation	Sub theme Satisfactory reimbursement Non-satisfactory reimbursement		
**Institutional factors and willingness to participate in CTs**	Main theme	Main theme:	Sub theme	
	Selection of CT participants	CT information	Duration at the clinic	
	Sub theme: Fair selection un fair selection of CT participants	Awareness of CTs Source of information about CTs	long period as a clinic patient short period as a patient	
**Barriers to CT participation**	Sub theme: Breach of	Sub theme: many tests conducted in CTs	Sub theme Myths about CTs	Sub theme Side effects of CT drugs
	Confidentiality lack of privacy	loss of blood	-Fear of CT drugs	-treatment failure -Death

Qualitative data suggests that a satisfactory compensation package and incentives were the most important motivation for participation. Two FGD participants observed:

*They treated us really well, they would give us something to eat, and you would not be in a hurry to go home because you would know that you were going to get something to eat* (R4 female FGD2).

*Transport is very important because they would give me 20,000 Ug shs. Sometimes I may have used 5000Ugshs for travelling. It becomes beneficial to me that I have remained with some money and I can buy a cup of milk* (R4 male FGD 4).

The health providers appeared to be aware of the various forms of motivation, but were particularly concerned about whether the financial reimbursement did not amount to coercion:

*We have seen that even the little bits of compensation [laughs] to a participant in research is making people more interested to participate and sometimes I begin to wonder if it’s not coercion hmm [laughs] (*KII 4, Female).

However, some of the FGD participants thought that the compensation they received during clinical trials was not satisfactory and expected more despite the wide range of benefits available, for example one participant argued:

*For each time you came they would give you transport to go back they would give [UGX] 10,000. Each time they would call us, they would give breakfast for those that came early and others lunch. Apart from that, there was nothing else we would get. That is what they would give us and each time they would draw blood, they would give biscuits and splash* (R11 female FGD 3).

Other participants positively perceived the benefits that accrued in non-monetary terms, that is, the auxiliary services provided to them during their participation in clinical trials. For example, one FGD respondent appreciated the apparent ‘special’ status accorded to clinical trial participants that ensured that they jumped the queues and were closely followed-up by the study team:

*They would even call us on the day we are supposed to return to remind us that on such and such a day, you are expected which doesn’t happen the other side (out of the study). They really showed us care. Many times, you come and are worried about the queue, you worry about the time you will leave and yet the other side (while on the study) they would look for your file and bring it to you. They used to do everything for us* (R8 male FGD 3).

Discrepancies were observed with respect to perception of time spent in the clinic. Respondents were willing to participate in drug treatment clinical trials because of shorter time spent at the clinic, especially because they jumped the patient waiting queues. However, the requirement for participants to make frequent visits to the clinic for monitoring was a barrier to their willingness to participate in the trials. The health providers appreciated this practical challenge:

*If you’re going to require a participant to return to the clinic thrice a month yet they are used to coming back once every 3 months, then they are not likely to take part in a clinical trial. If somebody is used to coming here and spending 30 min then after the clinical trial, they have to spend 4 h, or something then may not want to participate* (KII 5, male).

There appeared to be gender variations regarding the importance of medical tests. While female participants appreciated the free comprehensive medical tests and examinations they were subjected to, the males found them many and seemed not to appreciate their importance, and this was perceived as a barrier to participation in clinical trials. Notably, the medical tests provided to participants enrolled in clinical trials would otherwise have been unaffordable to them. A female respondent described the services thus:

*It really helped me because I did not have the capacity to pay for all those tests. They checked the heart, screened for cervical cancer, checked for diabetes, checked for hypertension because I wouldn’t have been able to run all those tests* (R4 female FGD3).

In contrast, a male FGD participant expressed concern about the many tests conducted, describing it as torture:

*Personally, when I was told about the research the very first time, I felt uncomfortable*. *Now, my thinking is that my body is tortured checking for this and the other, do you understand? They check for this and the other and you are afraid. My wife told me if you take the research drugs and you get so weak and down, where shall we go? That put me off.*

(R8 male, FGD4).

Two male participants who had never participated in clinical trials attributed their non-participation to lack of awareness of such opportunities, as they had never been approached to take part in any clinical trial.

*“Now like myself, I personally have never been told about research and I reject it” (*R7 male FGD1).

Furthermore, the second male FGD participant noted that even though he had never been approached to take part in clinical trials, he had previously heard about them and he was afraid of the uncertainty of likely risk.

*Like I told you, I have never been approached but in the beginning, but my wife was involved in a research here. She was able to complete because theirs was for 2 years, God helped and there was no problem at all (*R10 male, FGD4).

In addition, patients are more likely to participate in a treatment clinical trial if they believe that they will have an improved health status. One female participant mentioned the following when relating to improvement in her health status.

*When I came, I was in a very poor state*, *I wanted to find out what the problem was. All the other hospitals I went to said I had a mental problem and that’s why I joined (*R10 female FGD3).

Furthermore, patients were willing to participate in clinical trials if they believed they were the ultimate beneficiaries of trials through the development of a wide variety of drugs or lead to reduced pill burden. Another male FGD participant observed how patients had benefited from a wide variety of drugs from clinical trials.

*In the past, we used to take only one type of drug but now even at the dispensing window, each one is given a different type of drug* (R1 male FGD2).

While drug treatment clinical trials were appreciated for the opportunity to access a wide variety of drugs, the myths and fears associated with new drugs like treatment failure and death led to reduced willingness to participate in clinical trials. During FGDs, for example, some participants were aware of the processes involved in drug development, including trials done on animals such as monkeys. They saw clinical trials as an extension of drug development processes, and worried about being used as test animals, with some equating the human beings who take part in drug trials to monkeys.

*Among the white people they would try the drugs on monkeys but then now, you the black person when they start trying drugs on you, they perceive it so negatively*. *Some people out there say that if you go and participate in a clinical trial, they remain using you.* (R8 male FGD4).

Patients who have negative beliefs about clinical trials were likely to negatively influence those contemplating participation in clinical trials. A female FGD participant explained:

*I was in a line, we had come to pick our drugs but when one health worker came to explain to us about a research study, one lady said what; research! I don’t like them (researches) then I asked her why she didn’t like it and she said those drugs can bring you harm to your life and you may even die (*R6 female FGD3).

It was not only patients who expressed this concern. The health providers too identified the negative consequences of myths associated with the development of new drugs on participants’ willingness to get involved in clinical trials:

*“Myths, myths about new drugs they are associated with so many risks and this is a barrier to participation” (KII 3, female).*

The uncertainty about how their data will be used, and the fear that their identities maybe publicised, were other important issues discussed as barriers to willingness to participate in clinical trials. By saying “…. *they [clinical trials] remain using you”,* participant R8 above neatly articulated this fear and a particular sense of vulnerability that perhaps researchers exploited study participants, who are often unable to determine what happens after enrolling in a clinical trial. In relation to information management, participants appeared to believe that research information somehow usually gets published in the newspapers. There were several people who held this view, but the account of one male participant suggests that it was a generalised belief and fear that researchers tend to discuss their participants in the newspapers.

*When you talk about research, they [patients] have the thinking that they may take part and they may start writing about them and publish them in Newspapers. I also discussed with my wife and she said if you are the ones that they are going to research on, you will appear in Newspapers (R3 male FGD4).*

The selection process of participants is key in influencing willingness to participate in clinical trials. Selection processes that are considered fair are more likely to attract a bigger number of participants. One FGD male participant seemed to be aware of this fact and attributed it to the fact that he had been a clinic patient for a long time and thus more knowledgeable of how things are run in the clinic:

*What I know is that the way that they have been selecting people for research is fair. The time I have spent here, I have never seen things not moving well when we come to get drugs. I have never seen anything not going well so that gives me strength that the way things are done at IDI, they have a way of doing things well. That helps me not to get upset that sometimes I may be left behind because they look for their people and we remain (laughs). Because I see that the treatment we get here is equal* (R7 male FGD4).

## Discussion

In this urban population of people living with HIV, we found that the willingness to participate in clinical trials was high. Quantitative data found that willingness to participate in drug treatment clinical trials was strongly associated with monetary benefits that accrued from participation and a belief that they received a special status during clinical trials. This means that perception of a satisfactory compensation package and special status accorded in treatment clinical trials influences willingness to participate in clinical trials.

Additionally, qualitative data suggests that provision of meals, compensation for time and transport reimbursement influenced participation in clinical trials. Participants who perceived the compensation package and incentives as satisfactory were not in a hurry to go back home when they visited the clinic. This suggests that participants were extrinsically motivated, as found reported in other studies [18–20]. However, people who thought that the compensation package was not satisfactory were less likely to participate in clinical trials. Our findings contradict research done in Florida [21] and earlier in Uganda, which found that HIV research participation is not necessarily influenced by material benefits and [monetary] compensation [22]. However, this could be attributed to the differences in the population and settings where the research was conducted.

Both the Uganda National Guidelines and various international guidelines, such as the Declaration of Helsinki and CIOMS, state that compensation of participants enrolling in research should not be considered a research benefit, and should not present undue inducement to potential research participants [23–27]. However, Uganda National Council for Science and Technology has not put a cap on how much people should be compensated across different studies. It is estimated that over 80% of Ugandans live in abject poverty on less than one USD per day [28] and, therefore, any financial compensation for research participation could be seen as a substantial income, especially for HIV patients who tend to be poorer than the general population [29]. Undue inducement is a critical concept to the discussion on participants’ willingness to take part in HIV treatment clinical trials. This concept is controversial and can be conceived as acting under duress to make a decision that one would not otherwise have made [30]. The danger of undue inducements can be a real cause of concern for research participants. In the context of engaging poor vulnerable participants in clinical trials in HIV, monetary incentives paid out as compensation to participants, as well as other benefits offered, can be contentious since it is highly possible that participants will accept to take part due to receiving money regardless of the risks associated with such involvement. A key question in this debate is whether research participants should be compensated differently based on their income [31, 32] to minimise coercion or undue inducement but this too may not resolve the question of the most poor in resource limited settings, for whom any monetary compensation is far beyond their usual income. Researchers and ethics review boards will continue to confront this challenge, but in doing this, they must ensure that a balance between promoting the social good of research and respecting the dignity of human patients should be considered.

Both the quantitative and qualitative findings demonstrate that participants’ motivation to participate in HIV treatment clinical trials was driven by a belief that they were receiving better treatment and that they were being treated by some of the best physicians with the latest medication. This created confidence and boosted their esteem. Importantly it conveyed a sense of special status to the participant, increasing their motivation to participate in treatment clinical trials. This can be described as a form of extrinsic motivation. These findings are consistent with a study that indicated that being seen by good physicians and taking the latest drugs strongly motivated participation in clinical trials research [33, 34], and in particular, when a study drug is believed to possess health benefits [35–38]. Traditionally, in research ethics, there is a well-established dichotomy between being enrolled in a clinical study and receiving routine medical care. This distinction is usually articulated to prevent therapeutic misconception. This term is used to describe situations where a research subject fails to appreciate the distinction between the imperative of clinical research and of ordinary treatment, and therefore, inaccurately attributes therapeutic intent to research procedures [39]. This means that a participant enrolled in a study believes they are going to receive therapy and do not recognize that they are enrolled in a clinical research that may, for example, not provide any medical benefits, for instance if provided a placebo. Our study participants did not seem to appreciate this possibility; rather all of them appeared to believe that participating in a clinical trial came with many medical benefits. Such misconception can lead people to act against their own best interest and enrol in studies regardless of the potential risk. In a well-argued article, Gearhart examines this issue and noted that, indeed, there are cases where therapeutic misconception remains an ethical concern, such as when comparing an experimental treatment with placebo [40]. However, he argues that the traditional division between pure research and routine medical care might be blurring, as in fact, the whole point of clinical research is to assess a potential health benefit, and therefore we *should not be surprised when study participants ignore our disclaimers and assume there is, in fact, a good chance they will obtain a health benefit (p.g 1).*

Qualitative findings revealed that tests carried out during clinical trials motivated participation in trials. Previous research conducted by Whyte [41] among people living with HIV in Uganda has described the value that lay people attach to the importance of tests in the routine monitoring of treatment experience. Tests such as an HIV test, viral load and CD4 count, according to Whyte et al’s participants added new dimensions to bodily experiences; people related social situations and possibilities, for instance the hope to get a child, or even a partner. In our study, qualitative data found that participants appreciated the various tests conducted on them, although there appeared to be a gendered variation. While women particularly appreciated the tests and saw it as sign of special attention accorded to them by monitoring their health status for tests they could not have afforded on their own, some men did not seem to attach the same importance to the many tests and were concerned about the pricks and the amount of blood drawn. These qualitative findings are not conclusive. Future quantitative research should explore these issues.

Both the qualitative and quantitative results show that clinical trial participants appeared to receive special consideration from study staff who monitored their health status, provided regular communication, provided access to senior physicians and helped them jump the patient waiting queues. In this study, participants appeared to find great satisfaction with bypassing the queues and finding that clinicians were ready to review them. This meant that one spent less time during the clinic visits and were able to attend to other personal activities. Previous studies have shown similar findings and describe the importance of patients spending less time in the clinic [42, 43]. In addition, a sense of care and belonging was associated with receipt of regular communication on adherence and reminders about clinic appointments.

The qualitative findings revealed that in this HIV positive population, participants held with high regard issues of confidentiality and esteem. Patients were not comfortable taking part in clinical trials for fear that their privacy would be breached if they got published in Newspapers and their esteem affected if they were categorised as monkeys because CTs were used to test medicine on them which was a barrier to participation. This is a display of intrinsic motivation; the participants are concerned about their welfare regardless of incentives offered to them. Concerns about protecting the privacy of participant information, esteem, respect for persons which encompasses health information privacy and data anonymization are important motivators for willingness to participate in CTs [35, 37, 44].

Additionally, both quantitative and qualitative research findings demonstrate [38] that a fair selection process for research participants coupled with information and awareness about clinical trials are important and have an influence willingness to participate in HIV treatment clinical trials. Similar research conducted on willingness to participate in clinical trials is in agreement with the our findings that showed that people who have a better understanding and knowledge of clinical trials are more likely to take part in treatment clinical trials [36, 45, 46].

Gender considerations were noted, while most of the men had never been approached, their spouses had prior experience with participation. This could be attributed to the busy schedules demonstrated by men who spend less time in the clinic.

The study results confirm what other scholars have documented in relation to extrinsically motivating participants in research. Our participants highly valued material and health rewards received during in clinical trials, as has been found elsewhere [15]. However, by identifying barriers such as lack of privacy and fear of breach of confidentiality and the perception that clinical trial participants are equivalent to test animals, which affects their esteem, suggests that participants are not always extrinsically motivated. Cherry refers to the value of intrinsic motivation in sustaining participation in an activity, and warns against the costs associated with external rewards [47]. Extrinsic motivation leads to higher enrolment of participants over a short period of time because of the rewards associated with participation. On the other hand, intrinsic motivation leads to an improvement in the retention rates in the long run. Participant understanding of the relevance of clinical trials to individuals and their contribution to the community, is an influencing factor for willingness to participate in clinical trials [48].

Three key limitations of our study were as follows; first is a small sample size which was not attained because despite the study site being a large clinical trial centre, participant enrolment into this study was not feasible as only specialized patients are currently only being reviewed at IDI. Nevertheless, the use of a mixed methods approach ensured that qualitative data provided in depth insights about willingness to participate in clinical trials and demonstrated that most participants were willing to participate in clinical trials. Secondly, the findings are limited to HIV positive population in treatment clinical trials. This limits generalizability of our results to HIV treatment clinical trials. However, findings in this study will help address the challenges involved in undertaking clinical trials in this population. Thirdly, compensation expectation comparisons were not clear for a once-off clinical trial involving a single dosage and one follow up visit to those on a 5 year study involving numerous visits over the course of 5 years. Never the less the study provides useful information on compensation for consideration in treatment clinical trials for single dosage and follow up visits.

## Conclusion

In conclusion, our study indicates that individuals are extrinsically motivated to participate in HIV treatment clinical trials by the perceived rewards such as a fair compensation package and additional benefits. Investigators should provide the rationale of conducting clinical trials to participants with a focus on intrinsic motivation as this will aid recruitment in the long run. Study investigators, researchers should leverage this willingness to facilitate enrolment in clinical trials and pay attention to how participants’ concern for benefits may override the need to understand study procedures and risks. Further research should explore patient willingness across diverse settings. Future studies should compare compensation expectations for participants in a once off CT to those with numerous visits over a longer period.

## Data Availability

FGD study guide, KII guide and questionnaire are attached in additional file 1. Source data is in the form of a data base and a recorded voice which could break anonymity. All KII and FGD have been anonymised, transcribed and translated into English. Due to the sensitivity attached to this material it will not be available online. However, this anonymised data may be available on reasonable request from the author.

## Abbreviations

HIV: Human immune virus
IDI: Infectious Diseases Institute
FGD: Focus group discussion
KII: Key informant interview
GLM: Generalised linear model
CT: Clinical trial
PLHIV: People living with HIV

## References

[1] UPHIA (2017). Uganda population based HIV impact assessment.

[2] Ondoa C (2014). *The HIV & AIDs Uganda country progress report 2014*.

[3] Mafigiri R, Matovu JK, Makumbi FE, Ndyanabo A, Nabukalu D, Sakor M (2017). HIV prevalence and uptake of HIV/AIDS services among youths (15–24 years) in fishing and neighboring communities of Kasensero, Rakai District, south western Uganda. BMC Public Health.

[4] Haberer JE, Sabin L, Amico KR, Orrell C, Galárraga O, Tsai AC, Vreeman RC, Wilson I, Sam‐Agudu NA, Blaschke TF, Vrijens B. Improving antiretroviral therapy adherence in resource‐limited settings at scale: a discussion of interventions and recommendations. Journal of the International AIDS Society. 2017;20(1):21371. 10.7448/IAS.20.1.21371 PMC5467606 28630651

[5] Chu SH, Kim EJ, Jeong SH, Park GL (2015). Factors associated with willingness to participate in clinical trials: a nationwide survey study. BMC Public Health.

[6] Corbie-Smith G, Thomas SB, Williams MV, Moody-Ayers S (1999). Attitudes and beliefs of African Americans toward participation in medical research. J Gen Intern Med.

[7] Hussain-Gambles M, Leese B, Atkin K, Brown J, Mason S, Tovey P. Involving South Asian patients in clinical trials. Health Technol Assess. 2004;8(42):1–09. 10.3310/hta8420 15488164

[8] Rikin S, Shea S, LaRussa P, Stockwell M (2017). Factors associated with willingness to participate in a vaccine clinical trial among elderly Hispanic patients. Contemp Clin Trials Commun.

[9] Mbunda T, Bakari M, Tarimo EA, Sandstrom E, Kulane A. Factors that influence the willingness of young adults in Dar es Salaam, Tanzania, to participate in phase I/II HIV vaccine trials. Global health action. 2014;7(1):22853. 10.3402/gha.v7.22853 PMC3936110 24572007

[10] Cummings B, Mengistu M, Negash W, Bekele A, Ghile T (2006). Barriers to and facilitators for female participation in an HIV prevention project in rural Ethiopia: findings from a qualitative evaluation. Cult Health Sex.

[11] Nyblade L, Singh S, Ashburn K, Brady L, Olenja J (2011). “Once I begin to participate, people will run away from me”: understanding stigma as a barrier to HIV vaccine research participation in Kenya. Vaccine.

[12] Ssali A, Poland F, Seeley J (2015). Volunteer experiences and perceptions of the informed consent process: lessons from two HIV clinical trials in Uganda. BMC Med Ethics.

[13] Ssali A, Nunn A, Mbonye M, Anywaine Z, Seeley J (2017). Reasons for participating in a randomised clinical trial: the volunteers' voices in the COSTOP trial in Uganda. Contemp Clin Trials Commun.

[14] Ryan RM, Deci EL (2000). Self-determination theory and the facilitation of intrinsic motivation, social development, and well-being. Am Psychol.

[15] Hennessey B, Moran S, Altringer B, Amabile TM (2015). Extrinsic and intrinsic motivation. Wiley encyclopedia of management.

[16] AL-Tannir MA, El-Bakri N, Abu-Shaheen AK (2016). Knowledge, attitudes and perceptions of Saudis towards participating in clinical trials. PLoS One.

[17] Fereday J, Muir-Cochrane E (2006). Demonstrating rigor using thematic analysis: A hybrid approach of inductive and deductive coding and theme development. Int J Qual Methods.

[18] Collins AB, Strike C, Guta A, Turje RB, McDougall P, Parashar S, McNeil R. “We’re giving you something so we get something in return”: perspectives on research participation and compensation among people living with HIV who use drugs. Int J Drug Policy. 2017;39:92–8. 10.1016/j.drugpo.2016.09.004 PMC5396839 27780116

[19] de Castro, L., & Teoh, C. (2012). Payment of research subjects, Ethical Issues in.

[20] Peter M. Ellis, Butow, P. N., Tattersall, M. H. N., Dunn, S. M., & Houssami, N. (2001b). Randomized clinical trials in oncology: understanding and attitudes predict willingness to participate. American society of clinical oncology. 10.1200/JCO.2001.19.15.3554 11481363

[21] Cook C, Mack J, Cottler LB (2018). Research participation, trust, and fair compensation among people living with and without HIV in Florida. AIDS Care.

[22] McGrath JW, Mafigiri D, Kamya M, George K, Senvewo R, Svilar G (2001). Developing AIDS vaccine trials educational programs in Uganda. J Acquir Immune Defic Syndr.

[23] Council for International Organizations of Medical Sciences (2002). International ethical guidelines for biomedical research involving human subjects. Bull Med Ethics.

[24] Uganda National Council for Science Technology (2014). *National guidelines for research involving humans as research participants*: Uganda National Council for Scince and Technology Kampala-Uganda.

[25] van Delden JJ, van der Graaf R (2017). Revised CIOMS international ethical guidelines for health-related research involving humans. Jama.

[26] World Medical Association (2013). World medical association declaration of Helsinki: ethical principles for medical research involving human subjects. Jama.

[27] World Medical Association. (2015). WMA Declaration of Helsinki—ethical principles for medical research involving human subjects. 2013. *Google Scholar*. 10.1001/jama.2013.281053 24141714

[28] UBOS (2010). Uganda national survey report.

[29] UAC (2017). Levels of HIV discrimination in Uganda.

[30] Huberman M (1999). The mind is its own place: the influence of sustained interactivity with practitioners on educational researchers. Harv Educ Rev.

[31] Klitzman R (2013). How IRBs view and make decisions about coercion and undue influence. J Med Ethics.

[32] Singer E, Couper MP (2008). Do incentives exert undue influence on survey participation? Experimental evidence. J Empir Res Hum Res Ethics.

[33] Miller FG, Brody H (2003). A critique of clinical equipoise: therapeutic misconception in the ethics of clinical trials. Hastings Cent Rep.

[34] Nurgat Z, Craig W, Campbell N, Bissett J, Cassidy J, Nicolson M (2005). Patient motivations surrounding participation in phase I and phase II clinical trials of cancer chemotherapy. Br J Cancer.

[35] Braunstein JB, Sherber NS, Schulman SP, Ding EL, Powe NR (2008). Race, medical researcher distrust, perceived harm, and willingness to participate in cardiovascular prevention trials. Medicine.

[36] Carman AS, Sautter C, Anyanwu JN, Ssemata AS, Opoka RO, Ware RE (2020). Perceived benefits and risks of participation in a clinical trial for Ugandan children with sickle cell anemia. Pediatr Blood Cancer.

[37] Halpern SD, Karlawish JH, Casarett D, Berlin JA, Townsend RR, Asch DA (2003). Hypertensive patients' willingness to participate in placebo-controlled trials: implications for recruitment efficiency. Am Heart J.

[38] Moorcraft SY, Marriott C, Peckitt C, Cunningham D, Chau I, Starling N (2016). Patients’ willingness to participate in clinical trials and their views on aspects of cancer research: results of a prospective patient survey. Trials.

[39] Rikin S, Shea S, LaRussa P, Stockwell M. Factors associated with willingness to participate in a vaccine clinical trial among elderly Hispanicpatients. Contemporary clinical trials communications. 2017;7:122-5. 10.1016/j.conctc.2017.06.010 PMC5898544 29696176

[40] Gearhart J. If research is therapeutic, Where’s the misconception? J Clin Res Best Practices. 2018;14(12).

[41] Whyte SR. *Second chances: surviving AIDS in Uganda*: Duke University Press; 2014.

[42] Bogart LM, Chetty S, Giddy J, Sypek A, Sticklor L, Walensky RP (2013). Barriers to care among people living with HIV in South Africa: contrasts between patient and healthcare provider perspectives. AIDS Care.

[43] Twimukye A, King R, Schlech W, Zawedde FM, Kakaire T, Parkes-Ratanshi R (2017). Exploring attitudes and perceptions of patients and staff towards an after-hours co-pay clinic supplementing free HIV services in Kampala, Uganda. BMC Health Serv Res.

[44] McGraw D, Greene SM, Miner CS, Staman KL, Welch MJ, Rubel A (2015). Privacy and confidentiality in pragmatic clinical trials. Clin Trials.

[45] Buchbinder SP, Metch B, Holte SE, Scheer S, Coletti A, Vittinghoff E (2004). Determinants of enrollment in a preventive HIV vaccine trial: hypothetical versus actual willingness and barriers to participation. JAIDS J Acquired Immune Deficiency Syndromes.

[46] Ellis PM, Butow PN, Tattersall MH, Dunn SM, Houssami N (2001). Randomized clinical trials in oncology: understanding and attitudes predict willingness to participate. J Clin Oncol.

[47] Cherry K (2016). What is intrinsic motivation.

[48] Vansteenkiste M, Lens W, Deci EL (2006). Intrinsic versus extrinsic goal contents in self-determination theory: another look at the quality of academic motivation. Educ Psychol.

